# Practice Standards in International Medical Departments of Public Academic Hospitals in China: Cross-Sectional Study

**DOI:** 10.2196/53898

**Published:** 2024-05-13

**Authors:** Yaxu Zhou, Ying Zhou, Di Xu, Jie Min, Yu Du, Qi Duan, Wen Bao, Yingying Sun, Huiqin Xi, Chunming Wang, Evelyne Bischof

**Affiliations:** 1 Finance Department Renji Hospital Shanghai Jiaotong University School of Medicine Shanghai China; 2 Smart Hospital Development Department Renji Hospital Shanghai Jiaotong University School of Medicine Shanghai China; 3 International Medical Service Renji Hospital Shanghai Jiaotong University School of Medicine Shanghai China; 4 Nursing Department Renji Hospital Shanghai Jiaotong University School of Medicine Shanghai China; 5 Department of Oncology and Clinical Cancer Center State Key Laboratory of Oncogenes and Related Genes Shanghai Cancer Institute Shanghai China; 6 Department of Oncology Reni Hospital, School of Medicine Shanghai Jiao Tong University Shanghai China

**Keywords:** patients, international medical service, demand, satisfaction, strategy, health care optimization, smart hospital

## Abstract

**Background:**

Improving health care in cities with a diverse, international population is crucial for ensuring health equity, particularly for foreigners facing challenges due to cultural and language barriers. This situation is especially relevant in China, a major destination for expatriates and travelers, where optimizing health care services and incorporating international standards in the public sector are vital. Achieving this involves understanding the operational details, cultural and linguistic nuances, and advancing medical digitalization. A strategic approach focusing on cultural competence and awareness of health care systems is essential for effectively navigating health care for foreigners and expatriates in China.

**Objective:**

The aim of this study was to perform an in-depth analysis of the subjective and objective experiences of local and international patients in public hospitals in China to provide a basis for enhancing the medical experience of all patients.

**Methods:**

A structured questionnaire was provided to patients at an international outpatient service of a top-tier university hospital in China. Qualitative analysis of the survey responses was performed to methodically categorize and analyze medical treatment, focusing on patient demand and satisfaction across four main category elements (“high demand, high satisfaction”; “high demand, low satisfaction”; “low demand, high satisfaction”; and “low demand, low satisfaction”), enabling a detailed cross-sectional analysis to identify areas for improvement.

**Results:**

Elements falling under “high demand, high satisfaction” for both Chinese and international patients were primarily in the realms of medical quality and treatment processes. In contrast, elements identified as “high demand, low satisfaction” were significantly different between the two patient groups.

**Conclusions:**

The findings highlight the importance of systematic, objective research in advancing the quality of international health care services within China’s leading academic medical centers. Key to this improvement is rigorous quality control involving both patients and providers. This study highlights the necessity of certifying such centers and emphasizes the role of digital platforms in disseminating information about medical services. This strategy is expected to cater to diverse patient needs, enhancing the overall patient experience. Furthermore, by developing comprehensive diagnosis and treatment services and highlighting the superior quality and costs associated with international health care, these efforts aim to foster a sense of belonging among international patients and increase the attractiveness of China’s medical services for this demographic.

## Introduction

Improving health care processes to ensure equality in international metropoles has recently become a widely discussed challenge [1]. For foreigners living or traveling abroad, navigating the health care system presents unique challenges, especially when there are significant cultural and language differences. Given China’s substantial role as a global hub for expatriates and travelers, optimizing health care and establishing international service in the public sector are essential. This process requires a high-level understanding of operational intricacies, cultural and linguistic skills, as well as a great extent of medical digitalization [1-3]. These factors collectively necessitate a strategic approach to health care navigation for foreigners in China, underscoring the importance of cultural competence and systemic awareness in international medical settings [4].

Medical tourism represents a dynamic and growing sector within the global tourism and health care industries, characterized by individuals traveling across international borders to access medical treatments that may be unavailable, more costly, or have longer waiting times in their home countries [5]. In China, the medical tourism landscape is distinguished by its integration of traditional Chinese medicine (TCM) with modern health care services, offering a unique proposition to medical tourists [6]. China has seen significant growth in this sector, facilitated by its advancements in medical technology, the global reputation of its health care institutions, and government support for health care development [7]. The presence of high-quality health care services at relatively lower costs compared to those available in Western countries makes China an attractive destination for medical tourists seeking both conventional treatments and TCM therapies, leading to a significant growth in the number of foreign patients in hospitals [7]. Moreover, China’s status as a host for a large expatriate population adds another dimension to its medical tourism sector. Expatriates, alongside international tourists, contribute to the demand for high-quality and culturally sensitive health care services. This necessitates the adjustment of treatment offerings and health care service quality to cater to the diverse preferences and expectations of both medical tourists and the expatriate community residing in China. Ensuring the provision of health care that aligns with international standards and cultural sensitivities is crucial for maintaining China’s competitiveness as a preferred destination for medical tourism, as well as for fostering inclusivity for the expatriates.

In China, grade-3A hospitals represent the highest level within the three-tier hospital classification system, characterized by their extensive capacity for specialist health services, medical education, and scientific research [7]. Public hospitals, as the backbone of the Chinese health care system, integrate the best global practices and advanced digital infrastructures. Shanghai’s foreigner-oriented medical service institutions can be divided into three categories: an international medical service in a public hospital, a fully independent general hospital or clinic, or a “special medical care” sector in public hospitals [6,8]. The high quality of treatment for international patients in public hospitals, as evidenced by their increasing numbers, emphasizes accessibility, quality, and multidisciplinary expertise. In Shanghai, 61.6% of all foreign patients were treated in public hospitals between 2016 and 2018 [9,10].

International medical services in Shanghai public 3A-class hospitals have become the preferred choice for both Chinese and foreign patients because of the multidisciplinary excellence in clinical, educational, and scientific resources [11].

The Shanghai Renji University Hospital (hereafter referred to as Renji Hospital), one of the top three public hospitals in Shanghai, officially opened an international outpatient service in October 2015. This is the first international outpatient service platform in China that has passed the world-renowned international certification, assessment, and risk management Det Norske Veritas (DNV) certification for 3 consecutive years. Therefore, we selected Renji Hospital as a case study for a comprehensive cross-sectional analysis of the strategies employed and the outcomes achieved to enhance Shanghai’s global health care brand and, by extension, China’s international medical services framework.

Through a commitment to providing high-quality health care services to international patients, focused on understanding and meeting their specific needs and expectations, Chinese medical services embrace the importance of cultural sensitivity and cross-cultural communication. In 2022, the “14th Five-Year Plan” for Shanghai’s Health and Health Development was officially released, aiming at transforming Shanghai into an Asian “Medical City of Health” [12]. The strategy involves improving the health care service level, building a high-quality medical system, and incorporating an integrated and intelligent medical service that will ultimately transform the national public health system. At the same time, these efforts will increase the attraction of patients from neighboring countries and regions to come to Shanghai for medical treatment and enhance its international image [12].

## Methods

### Research Objective

This study was designed to evaluate and assess patient demands and satisfaction across various medical service elements. The aim of the study was to identify key areas of patient demand and satisfaction, particularly focusing on the international medical outpatient services. Analysis of the survey responses can help to provide guidelines on improving the medical experience for both local and international patients. Furthermore, these guidelines will serve as a tool for enhancing Shanghai’s quality of international patent care, thereby contributing to the city’s growth as China’s medical tourism hub.

The study was conducted in the international outpatient service of Renji Hospital. Recruitment for the study occurred over a 6-week period from February to March 2020. The selection process involved a stratified random sampling technique to ensure a representative sample of the international outpatient population attending the international outpatient service. Patients were approached by trained research coordinators who were fluent in both Mandarin and English, allowing for clear communication regarding the purpose of the study and the nature of the questionnaire. Inclusion criteria stipulated that participants must be international outpatients 18 years or older who had visited the international outpatient service within the past year.

Upon agreeing to participate, individuals were provided with the questionnaire in either English or Mandarin, based on their preference. To mitigate the potential for selection bias and to ensure privacy, questionnaires were completed anonymously in a designated area within the international outpatient service waiting room, with research coordinators available to assist with any queries.

### Ethical Considerations

The Ethical Committee of Renji Hospital exempted this study from the requirement of official approval since it was survey-based with voluntary participation. Patients were informed that participation was voluntary, with no incentives offered, and that their care would not be affected by their decision of whether to participate. The questionnaires were anonymous. The participants were informed about the nature of the research. This process highlighted that they could withdraw at any time without any consequences. The participants were not financially compensated for their participation.

### Questionnaire Design

Questionnaires were distributed before the medical treatment. The first section asked the patients to provide basic sociodemographic and general information, including customer category (first or return visit), sex, nationality, age, education level, payment method, and residence history in Shanghai.

To measure patients’ expectations (demand) and satisfaction from the medical service, an expert consortium designed a questionnaire drawing upon validated surveys from both local and international contexts related to hospital evaluation and patient satisfaction [13,14], as well as studies on the medical needs and satisfaction of foreign or commercially insured patients [15,16]. The questionnaire focused on five critical dimensions of health care provision: medical quality, physical environment, quality service, medical treatment process, and institutional qualification. Each dimension was further broken down into a total of 30 specific medical treatment aspects (eg, safety and efficiency), labeled Y1-Y30, to ensure a thorough evaluation.

The same questionnaire was distributed to the patients twice: before and after the visit. Before the visit, patients were asked to attribute their subjective importance to each of the categories showcasing their expectations (demand). After the visit, the patients were provided the questionnaire again, asking for the extent to which their demands were met (satisfaction).

The respondents’ demand scores for the 30 medical elements were compiled using the Likert 5-level scale method. Cronbach α was used for assessment of content validity to test the reliability and validity of the questionnaire. The Cronbach α coefficient was 0.964, confirming good structural reliability of the questionnaire. 

## Results

### Response Rate

Of the 200 questionnaires distributed, 180 were retrieved, yielding a high return rate of 90%. The responses underwent a preliminary screening to validate the completeness and consistency of the data, resulting in 171 questionnaires deemed valid for analysis. This led to an effective response rate of 94.4%, indicating robust engagement and reliability of the data collected. The high response rate may be reflective of the strong patient engagement and satisfaction with the services at the international outpatient service, which is consistent with the facility’s patient-centered approach to health care delivery.

### Sociodemographic and Core Characteristics of the Respondents

The basic information of the respondents is summarized in Table 1. Among the total 171 respondents, there were 92 Chinese patients and 79 foreign patients. The respondents were mainly patients seeking a second opinion, accounting for more than 60% of the sample. The proportion of men and women was similar, with 72.6% of the respondents under the age of 50 years. Overall, 91.3% of the respondents had received undergraduate education or above. Commercial insurance direct payment was the main payment method, accounting for 68.4% of the sample, and 75.4% of the respondents had been living in Shanghai for more than 3 years.

**Table 1:** Demographic and socioeconomic characteristics of the study participants (N=171).

Characteristics		Participants, n (%)
**Nationality**		
	Chinese	92 (53.8)
	Foreign	79 (46.2)
**Category**		
	First visit	66 (38.6)
	Return visit	105 (61.4)
**Sex**		
	Male	81 (47.4)
	Female	90 (52.6)
**Age (years)**		
	<20	2 (1.2)
	20-29	11 (6.4)
	30-39	62 (36.3)
	40-49	49 (28.7)
	50-59	43 (25.1)
	60-69	3 (1.8)
	≥70	1 (0.6)
**Education level**		
	High school	10 (5.8)
	Undergraduate	74 (43.3)
	Master’s	69 (40.4)
	Doctorate	13 (7.6)
	Other	5 (2.9)
**Payment method**		
	Self-pay	32 (18.7)
	Direct payment of commercial insurance	117 (68.4)
	Paid by commercial insurance company who settles the claim	22 (12.9)
**Shanghai residence history**		
	≤3 months	7 (4.1)
	3 months to 1 year	10 (5.8)
	1-3 years	25 (14.6)
	>3 years	129 (75.4)

### Demand Scores of Chinese and Foreign Patients

The average score of the respondents’ demand for each element was 4.61 out of 5, with the highest score found for medical quality (4.74), followed by medical treatment process (4.66), physical environment (4.59), institutional qualification (4.58), and quality service (4.47). Through single-factor analysis at an α level of .05, the difference in the scores of demand degree of patients of different nationalities in each dimension was found to be statistically significant. The scores of each item are shown in Table 2.

**Table 2:** Comparative analysis of demand scores among Chinese and foreign outpatients across five dimensions.

Dimension and items			All patients (N=171), mean (SD)		Chinese patients (n=92), mean	Foreign patients (n=79), mean
**Medical quality**			4.74 (0.40)			
	Y1: Attention paid to patient safety	4.90 (0.37)		4.92		4.87
	Y2: Effective clinical diagnosis and treatment	4.89 (0.39)		4.93		4.85
	Y3: Hospital infection management	4.83 (0.46)		4.80		4.86
	Y4: Attention paid to customer participation	4.63 (0.69)		4.68		4.57
	Y5: Attention paid to customer feedback	4.60 (0.73)		4.78		4.39
	Y6: Reasonable medical expenses	4.60 (0.71)		4.77		4.39
**Medical treatment process**			4.66 (0.48)			
	Y7: Convenient visit process	4.82 (0.45)		4.95		4.68
	Y8: Convenient and fast appointment	4.74 (0.58)		4.93		4.51
	Y9: Convenient payment	4.67 (0.66)		4.77		4.56
	Y10: Postdiagnosis follow-up service	4.61 (0.62)		4.62		4.59
	Y11: Smooth connection of referral	4.56 (0.74)		4.84		4.23
	Y12: Guided and accompanied services	4.53 (0.81)		4.75		4.28
**Physical environment**			4.59 (0.59)			
	Y13: Complete medical equipment	4.81 (0.48)		4.91		4.68
	Y14: Comfortable treatment environment	4.74 (0.53)		4.84		4.62
	Y15: Complete emergency facilities	4.71 (0.68)		4.85		4.56
	Y16: Efficient and convenient transportation	4.51 (0.88)		4.70		4.30
	Y17: Convenient and fast parking	4.18 (1.19)		4.51		3.78
**Institutional qualification**			4.58 (0.48)			
	Y18: Hospital brand reputation	4.81 (0.42)		4.89		4.72
	Y19: Hospital specialist ranking	4.77 (0.47)		4.88		4.65
	Y20: Insurance network hospital	4.65 (0.71)		4.74		4.56
	Y21: International certification	4.56 (0.69)		4.54		4.57
	Y22: Organization size	4.48 (0.81)		4.68		4.24
	Y23: Nature of hospital organization	4.23 (1.01)		4.42		4.01
**Quality service**			4.47 (0.70)			
	Y24: Protection of customer privacy	4.74 (0.70)		4.85		4.62
	Y25: Adequate information disclosure	4.70 (0.64)		4.74		4.65
	Y26: Attaches importance to informed consent	4.63 (0.77)		4.74		4.51
	Y27: Conforms to the medical care standards	4.49 (0.82)		4.55		4.42
	Y28: Multinational language service	4.32 (0.99)		4.15		4.52
	Y29: Carries out health education	4.31 (1.06)		4.36		4.25
	Y30: Respect for religious beliefs	4.13 (1.26)		4.35		3.87

### Satisfaction Scores of Chinese and Foreign Patients

The average score of respondents’ satisfaction with each element was 4.50 out of 5, with the highest scores obtained for medical treatment process (4.56), followed by medical quality (4.55), institutional qualification (4.54), physical environment (4.48), and quality service (4.34). Through single-factor analysis at an α level of .05, there were statistically significant differences in the satisfaction scores between groups. The scores of each item are shown in Table 3.

**Table 3:** Comparative analysis of satisfaction scores for Chinese and foreign outpatients across five domains.

Dimension and items		All patients (N=171), mean (SD)	Chinese patients (n=92), mean	Foreign patients (n=79), mean
**Medical quality**		4.55 (0.84)		
	Y1: Attention paid to patient safety	4.54 (1.16)	4.64	4.42
	Y2: Effective clinical diagnosis and treatment	4.61 (1.05)	4.75	4.44
	Y3: Hospital infection management	4.65 (1.00)	4.83	4.46
	Y4: Attention paid to customer participation	4.55 (0.93)	4.72	4.35
	Y5: Attention paid to customer feedback	4.47 (1.145)	4.71	4.19
	Y6: Reasonable medical expenses	4.46 (1.04)	4.63	4.27
**Medical treatment process**		4.56 (0.66)		
	Y7: Convenient visit process	4.68 (0.66)	4.82	4.53
	Y8: Convenient and fast appointment	4.77 (0.55)	4.90	4.62
	Y9: Convenient payment	4.67 (0.76)	4.84	4.48
	Y10: Postdiagnosis follow-up service	4.16 (1.58)	4.26	4.04
	Y11: Smooth connection of referral	4.78 (0.67)	4.86	4.70
	Y12: Guided and accompanied services	4.31 (1.325)	4.38	4.23
**Physical environment**		4.48 (0.77)		
	Y13: Complete medical equipment	4.68 (0.61)	4.87	4.47
	Y14: Comfortable treatment environment	4.54 (1.18)	4.63	4.43
	Y15: Complete emergency facilities	4.69 (0.86)	4.75	4.62
	Y16: Efficient and convenient transportation	4.6 (0.83)	4.83	4.33
	Y17: Convenient and fast parking	3.9 (1.62)	4.23	3.52
**Institutional qualification**		4.54 (0.80)		
	Y18: Hospital brand reputation	4.76 (0.69)	4.92	4.57
	Y19: Hospital specialist ranking	4.58 (1.06)	4.70	4.44
	Y20: Insurance network hospital	4.50 (1.20)	4.68	4.28
	Y21: International certification	4.37 (1.35)	4.48	4.24
	Y22: Organization size	4.43 (1.24)	4.71	4.10
	Y23: Nature of hospital organization	4.63 (0.93)	4.82	4.41
**Quality service**		4.34 (1.05)	4.45	4.22
	Y24: Protects customer privacy	4.25 (1.38)	4.11	4.41
	Y25: Adequate information disclosure	4.43 (1.22)	4.54	4.29
	Y26: Attaches importance to informed consent	4.44 (1.23)	4.60	4.27
	Y27: Conforms to the medical care standards	4.46 (1.30)	4.67	4.20
	Y28: Multinational language service	4.07 (1.70)	4.22	3.90
	Y29: Carries out health education	4.32 (1.30)	4.43	4.18
	Y30: Respect for religious beliefs	4.47 (1.06)	4.59	4.33

### Cross-Sectional Analysis of Patients’ Demands and Satisfaction

**Figure 1:**
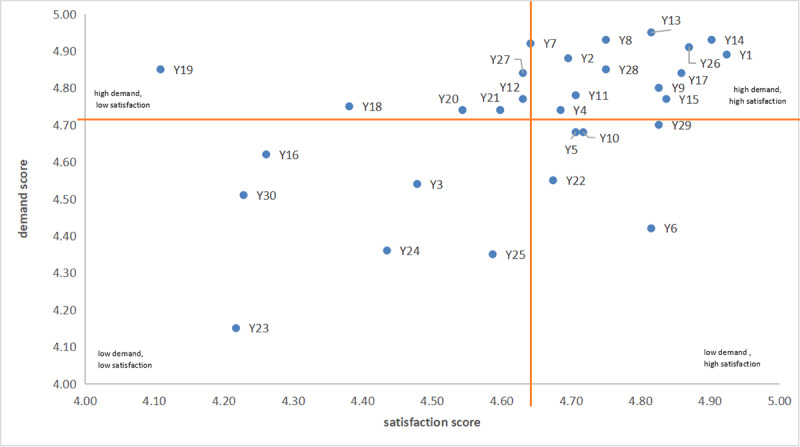
Quadrant chart of the evaluation of Chinese patients' needs based on a survey conducted at an urban hospital in China from January to December 2023. See Tables 2 and 3 for a description of each element Y1-Y30.

**Figure 2:**
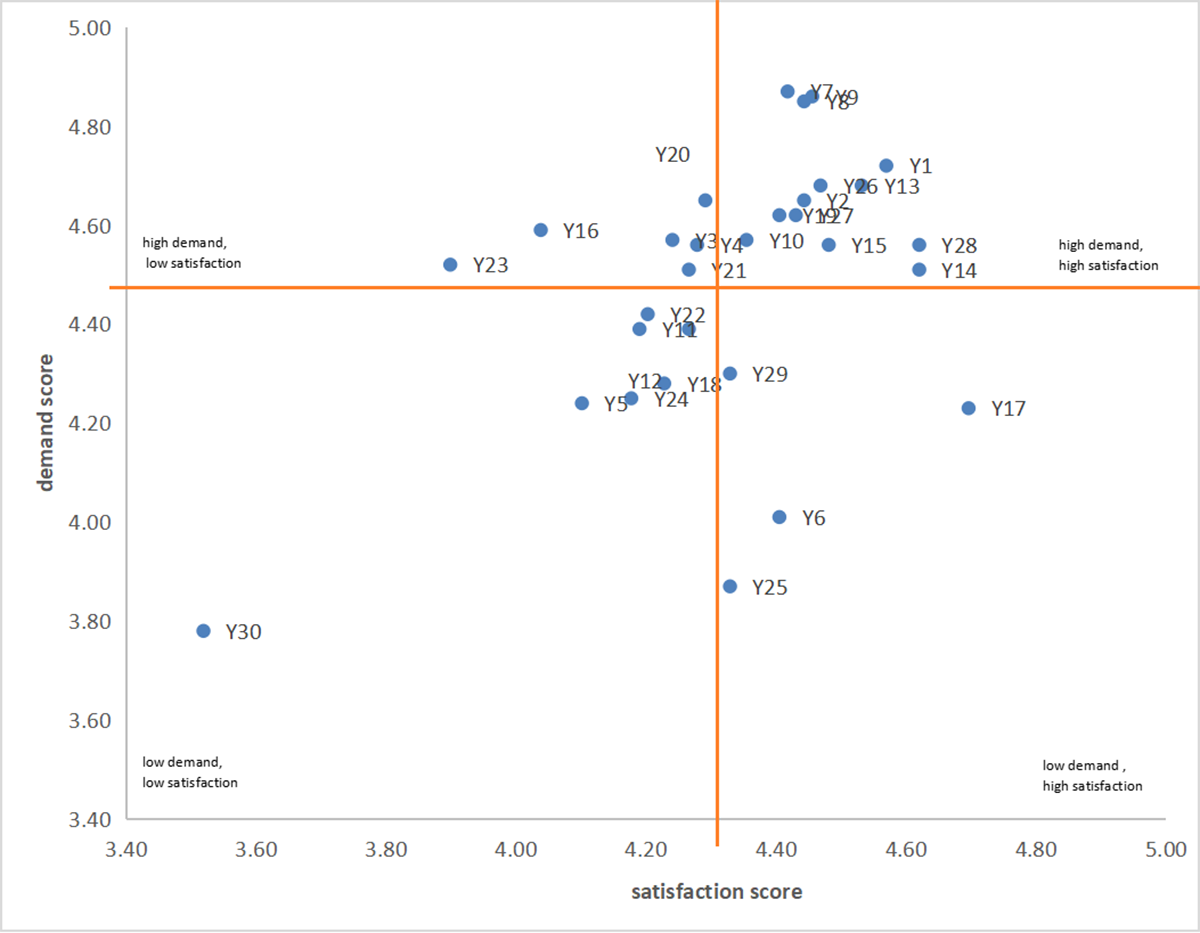
Quadrant chart of needs assessment of foreign patients based on a survey conducted at an urban hospital in China from January to December 2023. See Tables 2 and 3 for a description of each element Y1-Y30.

To visualize the relations between patients, demand, and satisfaction, scatterplot charts were plotted (Figures 1-2), where the x-axis represents the demand score and the y-axis represents the satisfaction score. The scatter chart was divided into four quadrants by taking the average satisfaction and demand scores as the boundary (as shown in the orange lines in Figures 1-2). The quadrants are as follows: Q1, “high demand, high satisfaction”; Q2, “high demand, low satisfaction”; “Q3, low demand, high satisfaction”; and Q4, “low demand, low satisfaction.”

### Demands and Satisfaction of Chinese Patients

For Chinese patients, medical treatment process and physical environment are perceived as equally important given that they were the most abundant categories in Q2 (high demand, low satisfaction). Institutional qualification scored the lowest, as Chinese patients’ high demands were not met for this category. The medical quality received outperformed Chinese patients’ demands, being the most abundant category in Q3 (low demand, high satisfaction). Regarding Q4 (low demand, low satisfaction), the quality service category was the most abundant, suggesting that Chinese patients did not expect and were not satisfied with the nonmedical services (Table 4).

**Table 4:** Four quadrants (Q1-Q4) of medical treatment elements for Chinese patients based on their demand and satisfaction levels.^a^

Category	Q1: high demand, high satisfaction	Q2: high demand, low satisfaction	Q3: low demand, high satisfaction	Q4: low demand, low satisfaction
Medical quality	Y1, Y2, Y4	N/A^b^	Y5, Y6	Y3
Medical treatment process	Y7, Y8, Y9, Y11	Y12	Y10	N/A
Physical environment	Y13, Y14, Y15, Y17	N/A	N/A	Y16
Institutional qualification	N/A	Y18, Y19, Y20, Y21	Y22	Y23
Quality service	Y26	Y27	Y29	Y24, Y25, Y30

^a^See Tables 2 and 3 for a description of each element Y1-Y30.

^b^N/A: not applicable.

### Demands and Satisfaction of Foreign Patients

For the foreign patients, the medical treatment process category performed the best as it had the most elements in Q1 (high demand, high satisfaction), suggesting that the high demands of international patients were met. However, Q1 had the most questions of all categories (13 questions), suggesting that foreign patients seek a comprehensive health care experience. Institutional qualification performed the most poorly, as it was the most abundant category in Q2 (high demand, low satisfaction), suggesting that the needs of foreign patients were not met. Quality service appears to have positively surprised foreign patients as it was the most abundant category in Q3 (low demand, high satisfaction). The question distribution in Q4 was uniform and the interpretation is inconclusive (Table 5).

**Table 5:** Four quadrants (Q1-Q4) of medical treatment elements for foreign patients based on their demand and satisfaction levels.^a^

Category	Q1: high demand, high satisfaction	Q2: high demand, low satisfaction	Q3: low demand, high satisfaction	Q4: low demand, low satisfaction
Medical quality	Y1, Y2	Y3, Y4	Y6	Y5
Medical treatment process	Y7, Y8, Y9, Y10	N/A^b^	N/A	Y11, Y12
Physical environment	Y13, Y14, Y15	Y16	Y17	N/A
Institutional qualification	Y19	Y20, Y21, Y23	N/A	Y18, Y22
Quality service	Y26, Y27, Y28	N/A	Y25, Y29	Y24, Y30

^a^See Tables 2 and 3 for a description of each element Y1-Y30.

^b^N/A: not applicable.

### Comparison of “High Demand, High Satisfaction” Points Between Chinese and Foreign Patients

The Chinese and foreign patients exhibited similar preferences and satisfaction levels regarding aspects categorized under “high demand, high satisfaction.” This suggests that certain aspects of health care services that are highly sought after and meet expectations well are relatively consistent across both patient groups.

In Q1, the scores to 9 questions overlapped between Chinese and foreign patients: attention paid to patient safety, effective clinical diagnosis and treatment, convenient visit process, convenient and fast appointment, convenience payment, complete medical equipment, comfortable treatment environment, complete emergency facilities, and attaches importance to informed consent. This suggests that both groups have high expectations regarding the quality of medical procedures and care inherent to standard health care; thus, both groups ranked these demands as being met with high satisfaction.

The high score of patients’ feelings regarding the prediagnosis appointment and postdiagnosis payment is likely due to the construction of Renji’s public hospital as an internet hospital and the implementation of mobile payment [17].

In Q1, there were 4 questions unique to foreign patients: multinational language service, conform to the medical care standards, hospital specialist ranking, and postdiagnosis follow-up service. The difference in demand for a multinational language service represents a reasonable distinction between Chinese and foreign patients given that its existence is tailored to foreign patients only and therefore there was no need for this category to be among the high demands of Chinese patients.

The unique questions for Chinese patients in Q1 included attention paid to customer participation, smooth connection of referral, and convenient and fast parking.

Both groups marked “hospital specialists ranking” as a high-demand point, but only foreign patients marked it as both high demand and high satisfaction, suggesting that international patients are more satisfied with the doctors’ rankings.

Complete medical equipment/emergency facilities refer to facilities having the necessary infrastructure for high-quality medical services [18,19]. Hospital brand reputation/hospital specialist ranking is the externalization of high medical quality [20]. Therefore, the core medical demand of Chinese and foreign patients in international clinics is medical quality. Based on medical quality, the demand for service provider equipment and specialist reputation is extended. It is suggested that the international medical service entities in the public context must consolidate the bottom line of medical quality, improve medical facilities and equipment, highlight the brand publicity centered on medical quality, and demonstrate the image and concept of “effective and safe” to patients.

### Comparison of “High Demand, Low Satisfaction” Points Between Chinese and Foreign Patients

In Q2, only two of the questions (insurance network hospital and international certification) overlapped between Chinese and foreign patients, which both belong to the institutional qualification category.

The aspects for which foreign patients had high demands and these expectations were not met included hospital infection management, attention paid to customer participation, efficient and convenient transportation, and nature of hospital organization. The high demands of Chinese patients were not met in the following aspects: guided and accompanied services, hospital brand reputation, hospital specialist ranking, and conform to the medical care standards. Interestingly, 4 out of 6 of these questions fall under the institutional qualification category.

To address areas of “high demand, low satisfaction,” it is crucial for international medical services to accelerate investment, develop strategies, and implement targeted improvements.

### Comparison of “Low Demand, High Satisfaction” Points Between Chinese and Foreign Patients

In Q3, two questions (carries out health education and reasonable medical expenses) overlapped between the Chinese and foreign patients; the low demand and high satisfaction for these aspects suggest that the services outperformed the expectations for both groups. This overlap suggests that both groups were positively surprised by the extent of health education and affordable prices at Renji Hospital.

The unique items in this quadrant for Chinese patients were attention paid to customer feedback, postdiagnosis follow-up service, and organization size. The unique items for the foreign patients were convenient and fast parking and adequate information disclosure.

It can be assumed that Chinese patients did not demand for their feedback to be heard (Y5), but they were positively surprised that they were asked for it, whereas the responses to this question for the foreign patients landed in Q4 (low demand, low satisfaction). By contrast, foreign patients were positively surprised by the adequate information disclosure, whereas Chinese patients’ scores for this item landed in Q4.

Both groups were highly satisfied with the fast and convenient parking at the hospital; however, only the Chinese patients had a demand for this aspect of service.

### Comparison of “Low Demand, Low Satisfaction” Points Between Chinese and Foreign Patients

In Q4, only two questions overlapped between Chinese and foreign patients: protect customer privacy and respect for religious beliefs, suggesting that both groups see the hospital setting as a public space and their feelings of privacy should be enhanced.

The unique items in Q4 for the foreign patients were attention paid to customer feedback, smooth connection of referral, guided and accompanied services, hospital brand reputation, and organization size.

Both Chinese and foreign patients had low demand for the organization size; however, only the Chinese patients marked this item with high satisfaction scores. In addition, only the Chinese group had a high demand for guided accompany services, although both groups rated this item as a low-satisfaction category. Both groups rated hospital brand reputation as a low-satisfaction item, whereas this was only indicated as a high-demand item for the Chinese patient group.

The unique items in Q4 for Chinese patients were infection management, efficient and convenient transportation, nature of hospital organization, and adequate information disclosure.

## Discussion

### Principal Findings

This study represents the first comprehensive cross-sectional analysis of patient needs and satisfaction in an international outpatient service center at a top-tier academic hospital. Through cross-sectional analysis, 30 elements were divided into four categories: “high demand, high satisfaction”; “high demand, low satisfaction”; “low demand, low satisfaction”; and “low demand, high satisfaction.”

The analysis investigated a practical experience from the construction of the international outpatient service within a large top-tier academic Chinese public hospital. The findings provide valuable insights for the construction and enhancement of the international medical service model in Shanghai and are also applicable to the broader context of public health care in China.

Considering the limitations of public hospital resources, based on these findings, we can propose various strategies for continuous optimization and key improvement of the international outpatient experience.

### Strategies for Improving the Medical Experience of International Outpatients

#### Rely on the Advantages of Public Medical Quality With Third-Party International Certification to Deliver a Progressive Patient Experience

International medical service entities in the public context should take the traditional advantages of the medical quality management of public hospitals into consideration, combine the characteristics of international medical services, benchmark the international medical care standards, take the third-party international certification as the starting point, promote reform through evaluation, continue to consolidate the quality of medical services, and improve the sense of access of international outpatients to the elements of medical quality. Through the third-party international certification, the international outpatient service of Renji Hospital has established the organizational structure, system process, evaluation method, and continuous improvement measures of foreign-related medical quality and safety that are suitable for the framework of Chinese public hospitals. Accordingly, the medical quality and safety standards of the international outpatient service can become the benchmark of the quality of foreign-related medical service in China and can be used to further improve the diversified service quality and medical experience of large public hospitals.

#### Digitalization and Use of International Media Platforms

Internet platforms play a crucial role in hospitals’ information disclosure efforts [21,22]. For international medical entities operating in the public sphere, it is vital to establish distinct foreign-related medical service information platforms tailored to patient needs. These platforms should offer multilingual or bilingual Chinese-to-English options to ensure a high-quality and informed medical experience for international patients. However, despite the bilingual online system, it is still critical for the medical personnel and doctors to communicate effectively in foreign languages, as language barriers have been highlighted as a key deterrent for foreign patients seeking medical care in Shanghai [12,23].

The platforms should include comprehensive content covering qualification certification, specialty strengths, treatment processes, expert profiles, facility background, additional services, and pricing details. Timely updates on medical treatment guidance, international medical care schedules, insurance partnerships, and patient feedback channels are essential [24]. Additionally, disclosing hospital social responsibility information such as patient satisfaction and safety measures will enhance transparency and build patient trust. Renji Hospital exemplifies this practice by developing a bilingual internet platform for foreign-related medical services, elevating the online and offline medical experience, and meeting diverse health needs effectively.

#### Build the Service Chain of the Whole Diagnosis and Treatment Cycle

Providing high-quality medical care at an affordable price is a crucial characteristic of international medical services, especially within the public health care context. To prioritize patient satisfaction, it is important for international medical entities to not only fulfill basic diagnostic and treatment needs but to also offer a quality service and improve the nonmedical efficiency to enhance patients’ overall experience. Based on the findings of this study, the following measures are recommended.

First, it is necessary to establish a comprehensive service chain that covers the entire diagnosis and treatment cycle, including prediagnosis appointments, inpatient care, and postdiagnosis follow-up [25]. To achieve this, internet hospital platforms can be used to facilitate preappointment guidance and improve the information and visual guidance systems. Accompanying and guidance services should be provided to enhance interaction and improve efficiency. Various channels can be implemented for postdiagnosis follow-up, such as telephone, email, and internet hospital, to extend the service chain and foster patient engagement.

Second, it is essential to respect patients’ right to privacy [26]. This can be achieved by ensuring that each patient has access to a private consultation room and, if spatial constraints exist in the examination area, to use curtain screens or other methods to create separation. Staff should provide reassurance and clear explanations to make patients feel respected. In addition, the training and supervision of medical personnel should be enhanced, integrating the protection of patient privacy into their daily behaviors during diagnosis, treatment, and communication.

Third, it is necessary to maintain a comfortable and tidy international medical reception and treatment area [27]. This should be done in a manner that emphasizes the harmony and unity of both the physical environment and the decor. In addition, the area should be regularly maintained and cleaned, while guiding staff members to maintain a positive and professional attitude.

Implementing these measures will contribute to creating a favorable environment for international medical services, ensuring a balance between high-quality care and affordability while enhancing the overall patient experience.

#### Foster Inclusivity, Integration, and Internationality

Finally, a conducive environment for foreign-related medical services is one that combines local training and foreign expertise. First, it is crucial to enhance language training for staff to overcome language barriers. Implementing a comprehensive foreign-language training and assessment plan is recommended, ensuring that staff can proficiently master English expressions and medical terminology, pronounce the words accurately, respond promptly, and communicate effectively with foreign patients [28]. Second, there is a need to cultivate exceptional general practitioners and develop a medical service model that prioritizes “general practice before specialty,” aligning with the medical preferences of foreign patients [29]. It is advisable to explore and implement training, educational, and exchange programs for high-quality general practitioners, fostering practitioners with international medical attributes [30]. In this model, general practitioners would conduct initial diagnosis and treatment, accurately referring patients requiring further diagnosis and specialized treatment to relevant specialists based on professional judgment. This approach is better suited to the medical preferences of foreign patients and enhances their sense of belonging. Third, the introduction of renowned foreign experts can significantly contribute to improving services at an international hospital. Renji Hospital has established and improved standardized systems and processes for the practice registration, diagnosis, and treatment of foreign clinicians, gradually establishing a well-regulated management system. The hospital has successfully recruited two full-time doctors from the United States and Italy, as well as five part-time doctors from the United States, Canada, Germany, and Singapore, fostering a favorable practice of receiving foreign patients.

### Strengths and Limitations

The novelty of this study lies in the utilization of management tools to conduct a cross-sectional analysis of patient needs and satisfaction regarding medical treatment elements, leading to the accurate classification of 30 key elements. This approach enables practitioners and researchers involved in international medical service construction to identify crucial areas, implement targeted policies, enhance the efficiency of international medical services, and improve the overall patient experience.

However, it is important to acknowledge some limitations of this study. First, to emphasize key points, the strategies proposed may not encompass all aspects comprehensively. Second, the terms “low demand” and “low satisfaction” refer to relative values rather than absolute levels. In practical application, regular assessments of patients’ medical needs and satisfaction should be conducted, allowing for dynamic adjustments in focus areas. Third, the study was restricted to a single hospital’s international outpatient service and there is potential for response bias, as the participants who chose to respond may have different perspectives from those who did not participate. Additionally, the study period coincides with the onset of the COVID-19 pandemic, which may have influenced patient expectations and satisfaction levels.

### Conclusions

The findings from this study have broad implications for the improvement of international outpatient services within public hospitals. The results suggest that such entities must not only reinforce the foundation of medical quality but also enhance the visibility and accessibility of their services through better medical facilities, equipment, and brand communication. The study advocates for an increase in investment in areas of “high demand, low satisfaction,” such as language services and privacy protection, to provide targeted improvements. Emphasizing the importance of digital platforms for information dissemination and adopting a comprehensive approach to the diagnosis and treatment service chain can significantly improve patient experiences. As public hospitals continue to navigate the demands of a global patient base, these findings offer valuable insights for developing services that are not only of high quality but also culturally sensitive and patient-centered.

## Abbreviations

DNV: Det Norske Veritas
TCM: traditional Chinese medicine

## Data Availability

The data sets generated during and/or analyzed during this study are available from the corresponding author on reasonable request.
