# Correction: Practice Standards in International Medical Departments of Public Academic Hospitals in China: Cross-Sectional Study

**DOI:** 10.2196/60644

**Published:** 2024-05-27

**Authors:** Ying Zhou, Yaxu Zhou, Di Xu, Jie Min, Yu Du, Qi Duan, Wen Bao, Yingying Sun, Huiqin Xi, Chunming Wang, Evelyne Bischof

**Affiliations:** 1 Smart Hospital Development Department Renji Hospital Shanghai Jiaotong University School of Medicine Shanghai China; 2 Finance Department Renji Hospital Shanghai Jiaotong University School of Medicine Shanghai China; 3 International Medical Service Renji Hospital Shanghai Jiaotong University School of Medicine Shanghai China; 4 Nursing Department Renji Hospital Shanghai Jiaotong University School of Medicine Shanghai China; 5 Department of Oncology and Clinical Cancer Center State Key Laboratory of Oncogenes and Related Genes Shanghai Cancer Institute Shanghai China; 6 Department of Oncology Reni Hospital, School of Medicine Shanghai Jiao Tong University Shanghai China

In “Practice Standards in International Medical Departments of Public Academic Hospitals in China: Cross-Sectional Study” (JMIR Form Res 2024;8:e53898), the authors noted one error.

In the originally published article, the order of the authorship list was published as follows:

Yaxu Zhou, MPH; Ying Zhou, MPH; Di Xu, MPH; Jie Min, MPH; Yu Du, MPH; Qi Duan, MPH; Wen Bao, MPH; Yingying Sun, MPH; Huiqin Xi, RN; Chunming Wang, Prof Dr; Evelyne Bischof, Prof Dr

This has now been corrected to:

Ying Zhou, MPH; Yaxu Zhou, MPH; Di Xu, MPH; Jie Min, MPH; Yu Du, MPH; Qi Duan, MPH; Wen Bao, MPH; Yingying Sun, MPH; Huiqin Xi, RN; Chunming Wang, Prof Dr; Evelyne Bischof, Prof Dr

Consequently, affiliations 1 and 2 have been interchanged in order to align with the corrected authorship. They have changed from:

1 Finance Department, Renji Hospital, Shanghai Jiaotong University School of Medicine, Shanghai, China

2 Smart Hospital Development Department, Renji Hospital, Shanghai Jiaotong University School of Medicine, Shanghai, China

To the following:

1 Smart Hospital Development Department, Renji Hospital, Shanghai Jiaotong University School of Medicine, Shanghai, China

2 Finance Department, Renji Hospital, Shanghai Jiaotong University School of Medicine, Shanghai, China

The correction will appear in the online version of the paper on the JMIR Publications website on May 27, 2024, together with the publication of this correction notice. Because this was made after submission to full-text repositories, the corrected article has also been resubmitted to those repositories.

